# Sodium Valproate Alleviates Neurodegeneration in SCA3/MJD via Suppressing Apoptosis and Rescuing the Hypoacetylation Levels of Histone H3 and H4

**DOI:** 10.1371/journal.pone.0054792

**Published:** 2013-01-28

**Authors:** Jiping Yi, Li Zhang, Beisha Tang, Weiwei Han, Yafang Zhou, Zhao Chen, Dandan Jia, Hong Jiang

**Affiliations:** 1 Department of Neurology, Xiangya Hospital, Central South University, Changsha, China; 2 Neurodegenerative Disorders Research Center, Central South University, Changsha, China; 3 National Laboratory of Medical Genetics of China, Central South University, Changsha, China; 4 Department of Neurology & Institute of Translational Medicine at University of South China, the First People's Hospital of Chenzhou, Chenzhou, China; University of Iowa Carver College of Medicine, United States of America

## Abstract

Spinocerebellar ataxia type 3 (SCA3) also known as Machado-Joseph Disease (MJD), is one of nine polyglutamine (polyQ) diseases caused by a CAG-trinucelotide repeat expansion within the coding sequence of the *ATXN3* gene. There are no disease-modifying treatments for polyQ diseases. Recent studies suggest that an imbalance in histone acetylation may be a key process leading to transcriptional dysregulation in polyQ diseases. Because of this possible imbalance, the application of histone deacetylase (HDAC) inhibitors may be feasible for the treatment of polyQ diseases. To further explore the therapeutic potential of HDAC inhibitors, we constructed two independent preclinical trials with valproic acid (VPA), a promising therapeutic HDAC inhibitor, in both *Drosophila* and cell SCA3 models. We demonstrated that prolonged use of VPA at specific dose partly prevented eye depigmentation, alleviated climbing disability, and extended the average lifespan of SCA3/MJD transgenic *Drosophila*. We found that VPA could both increase the acetylation levels of histone H3 and histone H4 and reduce the early apoptotic rate of cells without inhibiting the aggregation of mutant ataxin-3 proteins in MJDtr-Q68- expressing cells. These results collectively support the premise that VPA is a promising therapeutic agent for the treatment of SCA3 and other polyQ diseases.

## Introduction

Spinocerebellar ataxia type 3 (SCA3), also known as Machado-Joseph Disease (MJD), is an autosomal dominant neurodegenerative disorder caused by a CAG-trinucelotide repeat expansion within the coding region of the *ATXN3* gene (also called the *MJD1* gene) [1]. SCA3 usually begins in adulthood, and it is characterized by a number of symptoms that include progressive cerebellar ataxia, dysarthria, dysphagia, oculomotor dysfunction and peripheral myotrophy [2, 3]. The glutamine stretch in the ataxin-3 protein ranges from 12 to 40 CAG repeats in normal individuals and expands from 52 to 86 repeat units in SCA3 patients [4]. There is an inverse correlation between the size of CAG repeats in SCA3 patients and their disease severity and the age at onset; the longer the repeat the earlier the age at onset [5]. Until now, no disease-modifying treatment for SCA3 has been established.

Recent studies suggest that transcriptional dysregulation may play an important role in the pathogenesis of the polyQ diseases. Transcriptional co-activators such as CREB-binding protein (CBP), p300/CBP-associated factor (PCAF), sperm-specific basic nuclear protein 1 (Sp1), and TBP-associated factor 4 (TAF_II_130) can be found in inclusion bodies and co-localized with polyglutamine proteins [6, 7]. Mutant proteins that contain the polyglutamine-rich domain inhibit histone acetylase activity of CBP/p300 though protein-protein interactions, and lead to cellular toxicity [8, 9]. Reversal of this hypoacetylation, which can be accomplished either by treatment with deacetylase inhibitors [10] or by over-expression of CBP, results in less neurodegeneration [11]. All of the above evidence supports the hypothesis that an imbalance in histone acetylation may be a key process leading to transcriptional dysregulation in polyQ diseases.

Histone acetylation and deacetylation is one form of posttranslational modifications of proteins, which regulates gene transcription by changing the compactness of nucleosome polymers [12]. The balance between histone acetylation and deacetylation, which is mediated by histone acetyltransferases (HATs) and histone deacetylases (HDACs), is usually well regulated, but it could be disorganized in polyQ diseases [13]. HDAC inhibitors, such as suberoylanilide hydroxamic acid (SAHA) [14], sodium butyrate (SB) [15] and trichostatin (TSA) [16], have been demonstrated to be effective in upregulating histone acetylation and in ameliorating motor impairments in mouse models of Huntington's disease (HD), spinal and bulbar muscular atrophy (SBMA) and dentatorubral-pallidoluysian atrophy (DRPLA). However, SAHA may not be a practical therapy, as it was found to be toxic at unacceptably low doses when tested in a phase I clinical trial for malignancies, such as leukopenia, thrombocytopenia, hypotension, acute respiratory distress, renal insufficiency, tumor-related pain and fatigue [17]. Although SB is less toxic than SAHA, this compound yielded beneficial effects within a narrow therapeutic dose window [18].

Valproic acid (VPA) is one of the HDAC inhibitors, and it has been used for decades to treat epilepsy and bipolar disorder. Its safety and tolerability has been clinically demonstrated [19]. In addition to functioning as an inhibitor of histone-deacetylases (HDAC) to regulate gene transcription, VPA has been shown to mediate neuronal protection by inducing the apoptosis-inhibiting gene *bcl-2* [20] and by inhibiting the activity of glycogen synthase kinase-3 [21]. Because of this established function, we expected that VPA could serve as a new treatment strategy for SCA3 and other polyQ diseases. In this study, we tested whether VPA treatment would be effective in both *Drosophila* and cell SCA3 models.

## Results

### MJDtr-Q78 transgenic *Drosophila* exhibits neurodegenerative phenotypes

**Figure 1:**
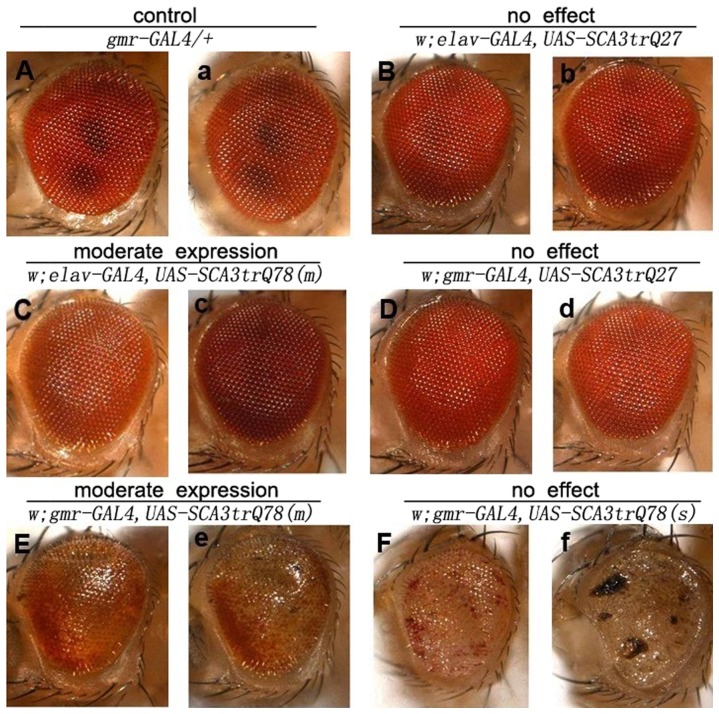
Expanded polyglutamine protein leads to eye degeneration of adult flies. (A–F) Dissecting microscopic images of the adult eyes of 1-day-old flies. (a–f) Dissecting microscopic images of the adult eyes of 15-day-old flies. The eyes of animals expressing MJDtr-Q27 protein (B, b and D, d) were identical to normal *Drosophila* phenotype (A and a), whereas those expressing moderate (C, c and E, e), and strong (F and f) *UAS-MJDtr-Q78* transgene insertions were mildly to severely disrupted, respectively. Moderate (E, e) lines showed a slight disruption of the regular, external lattice of the eye in young flies (E), and the eye morphology of these flies progressively degenerated during adult life (e). Strong lines (F and f) showed severely disrupted eye morphology with loss of pigmentation, collapsed and even appeared point-like necrosis during adult life.

**Figure 2:**
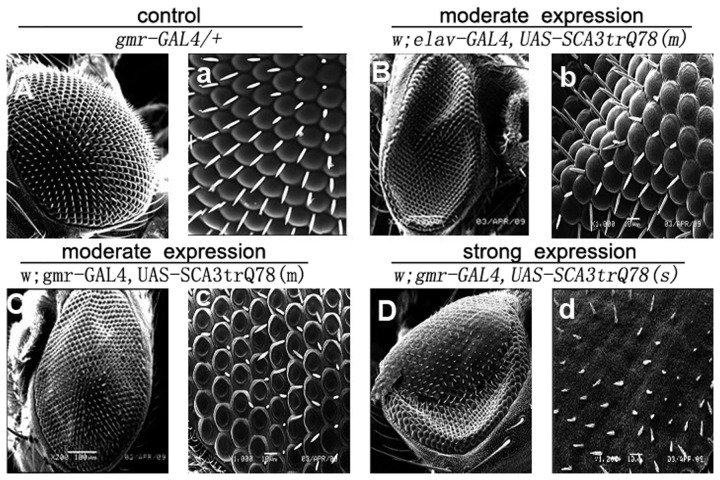
Expression of expanded polyglutamine protein disrupts eye morphology of adult flies. (A–D) Scanning election microscopic images of the eyes of 15-day-old adult flies (×200). (a–d) Scanning election microscopic images of the eyes of 15-day-old adult flies (×1000). Compared to normal *Drosophila* phenotype (A, a), moderate expression of MJDtr-78Q in eye caused mildly disrupted eye morphology (C, c), whereas strong expression of MJDtr-78Q in eye had quite deleterious effect on the eye phenotype (D, d).

We recreated a *Drosophila* model of SCA3 based upon a previously well-established *Drosophila* model [22]. We expressed the MJDtr-Q78 transgene both in the developing eyes and in neurons using the GAL4/UAS system [23], such that transgene expression could be directed specifically to the nervous system as well as to other tissues as desired. As a control, transgenic lines expressing MJDtr-Q27 protein were constructed. Compared to normal *Drosophila* phenotype (Fig. 1A, a, Fig. 2A, a), expression of the MJDtr-Q27 protein had no phenotypic effect (Fig. 1B, b, D, d). In contrast, expression of the MJDtr-Q78 protein produced deleterious phenotypes when expressed to select tissues. In flies with moderate expression, the developing eyes showed a mildly disrupted compound eye structure, with a loss of pigment cells visible on the edge at postnatal day 1 (Fig. 1E), which gradually evolved into the central area by postnatal day 15 (Fig. 1e, Fig. 2C, c). In flies with strong phenotypic expression, there was a greater loss of cell integrity, and the pigmentation of these flies was completely faded (Fig. 1F) with black point-like necrosis (Fig. 1f). These results were quite similar to previous study [22], in which MJDtr-Q78 targeted expression in the eye revealed late onset, progressive degeneration.

**Figure 3:**
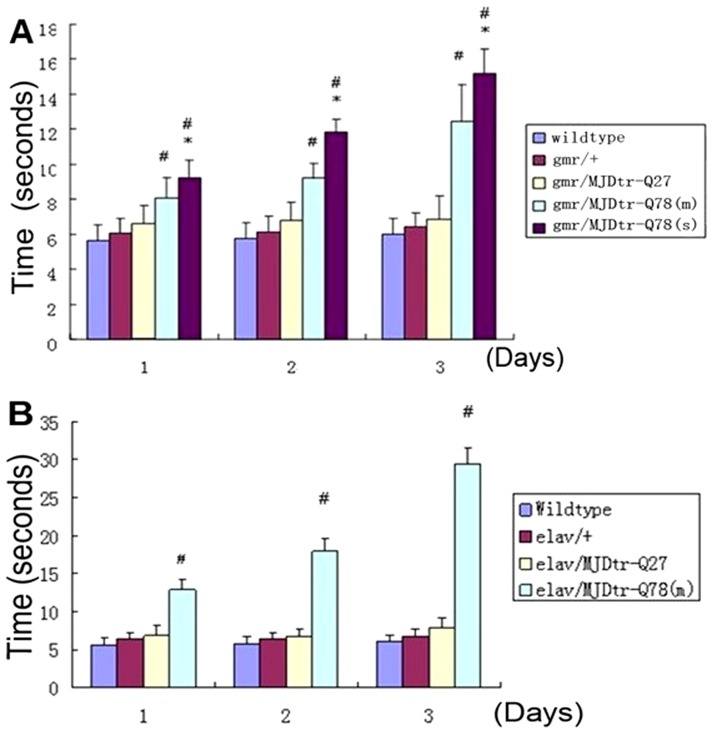
Differences in climbing ability of both *gmr-GAL4/UAS* (A) and *elav-GAL4/UAS* (B) SCA3 *Drosophila* model. X axis represents observing time:1 means the first day,2 means the fifth day, 3 means the fifteen day; # represents compared to wild-type flies, P<0.05; * represents compared to *gmr/MJDtr-Q78(m)*, P<0.05. Error bars represent S.E.M (n = 200).

**Figure 4:**
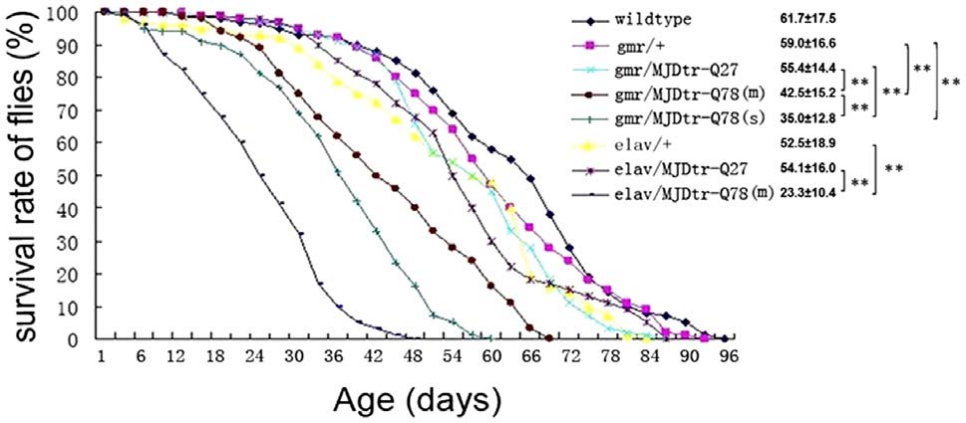
Expanded polyglutamine protein causes shortened life span in SCA3 transgenic *Drosophila* model. Differences in life span of female SCA3 transgenic *Drosophila* model were shown. As time went by, the life span of each transgenic flies shortened in a repeat length-dependent manner, and the most serious phenotypes can be seen when MJDtr-Q78 protein expressed in neurons. The mean life span and SD are shown, **: p<0.01 (n = 100).

The climbing ability of both fly strains was also impaired with time, all MJDtr-Q78-expressing flies showed decreased climbing ability, the most serious disabled climbing ability was observed in flies of genotype *ELAV-MJDtr-Q78 (m)* (Fig. 3B). Both *GMR-MJDtr-Q78* and *ELAV-MJDtr-Q78* flies displayed much shorter life span than controls; their mean life span were 42.5±15.2 and 23.3±10.4 days, respectively, whereas the controls had a mean life span of 59.0±16.6 days (Fig. 4). Moreover, *ELAV-MJDtr-Q78* flies were more seriously affected, their mean life span was the shortest in each group (Fig. 4). These results suggest that expression of MJDtr-Q78 protein causes neurodegeneration, moreover, it has more deleterious effects when selectively expressed in neurons. Our *Drosophila* model showed several neurodegenerative phenotypes that were similar to characteristics of human SCA3, suggesting that it was an incisive tool for exploring the therapeutic potential of VPA.

### VPA prevents against polyQ-induced eye depigmentation in MJDtr-Q78 transgenic *Drosophila*

**Figure 5:**
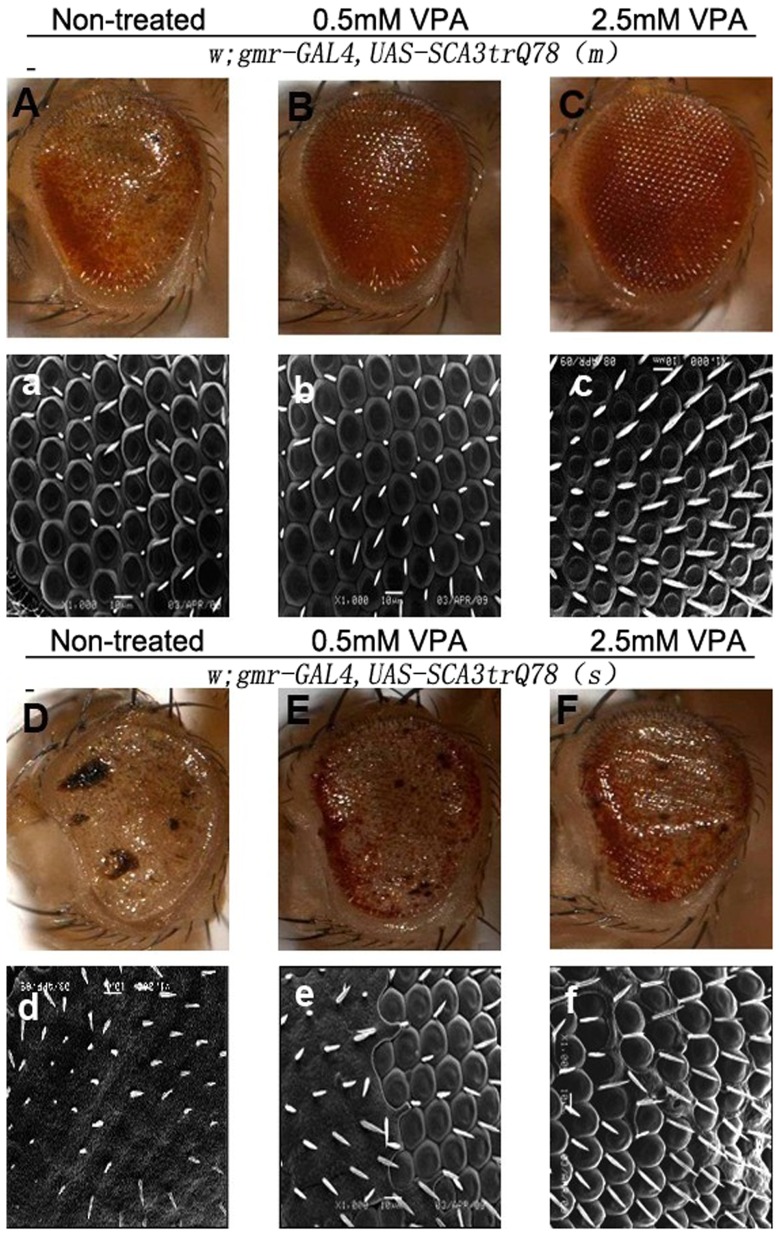
Valproic acid prevents against polyQ-induced eye depigmentation in SCA3 *Drosophila* model. (A–F) Dissecting microscopic images of the eyes of 15-day-old adult flies (×115). (a–f) Scanning election microscopic images of the eyes of 15-day-old adult flies (×1000). Non-treated flies expressing moderate (A and a) and strong (D and d) MJDtr-Q78 protein on eyes showed mildly to severely eye depigmentation, respectively. Treated with VPA at a dose of 0.5 mM (b, B and e, E) moderately prevented ommatidial disorganization, as seen by slightly less disrupted ommatidial structure compared with non-treated flies. Treated with VPA at a dose of 2.5 mM (C, c and F, f) strongly prevented eye depigmentation, with obviously increase of pigmentation, visible ommatidial pattern of the photorecepter rhabdomeres and nearly disappeared black point-like necrosis.

To test the therapeutic potential of VPA *in vivo*, daily doses of VPA at 0.5 mM, 1 mM, 1.5 mM, 2 mM and 2.5 mM were administered to SCA3 *Drosophila* model since after the flies reached adulthood. As previously mentioned, moderate and strong expressions of the MJDtr-Q78 protein in the eyes resulted in mild and severe eye depigmentation, respectively, including a loss of pigment cells, rough eyes collapse and disordered structured of the eyes (Fig. 5A, a, D, d). Intriguingly, after prolonged treatment with VPA for 15 days, eye depigmentation was partly prevented against in a dose-dependent fashion compared to non-treated controls. The number of pigment cells increased, the characteristic ommatidial pattern of the photoreceptor rhabdomeres was visible within the eye, and the presence of black point-like necrosis was reduced (Fig. 5). The most notable improvement was observed at a dose of 2.5 mM (Fig. 5C, c, F, f).

### VPA ameliorates loss of climbing ability in MJDtr-Q78 transgenic *Drosophila*

To further address the effect of VPA on the SCA3 phenotype, we tested whether VPA treatment affected the climbing ability of the SCA3 *Drosophila* model using the light-induced positive geotactic response [24]. We placed ten flies in to a 25 ml pipette. A fiber-optic lamp illuminated the pipette from the top. The flies were gently knocked to the bottom, and we measured the average time for the first 5 flies to cross a line 17.5 cm from the bottom. By this method, we evaluated the climbing ability of flies at the 5th and 15th days after eclosion from pupal cases, following treatment with different doses of VPA.

**Figure 6:**
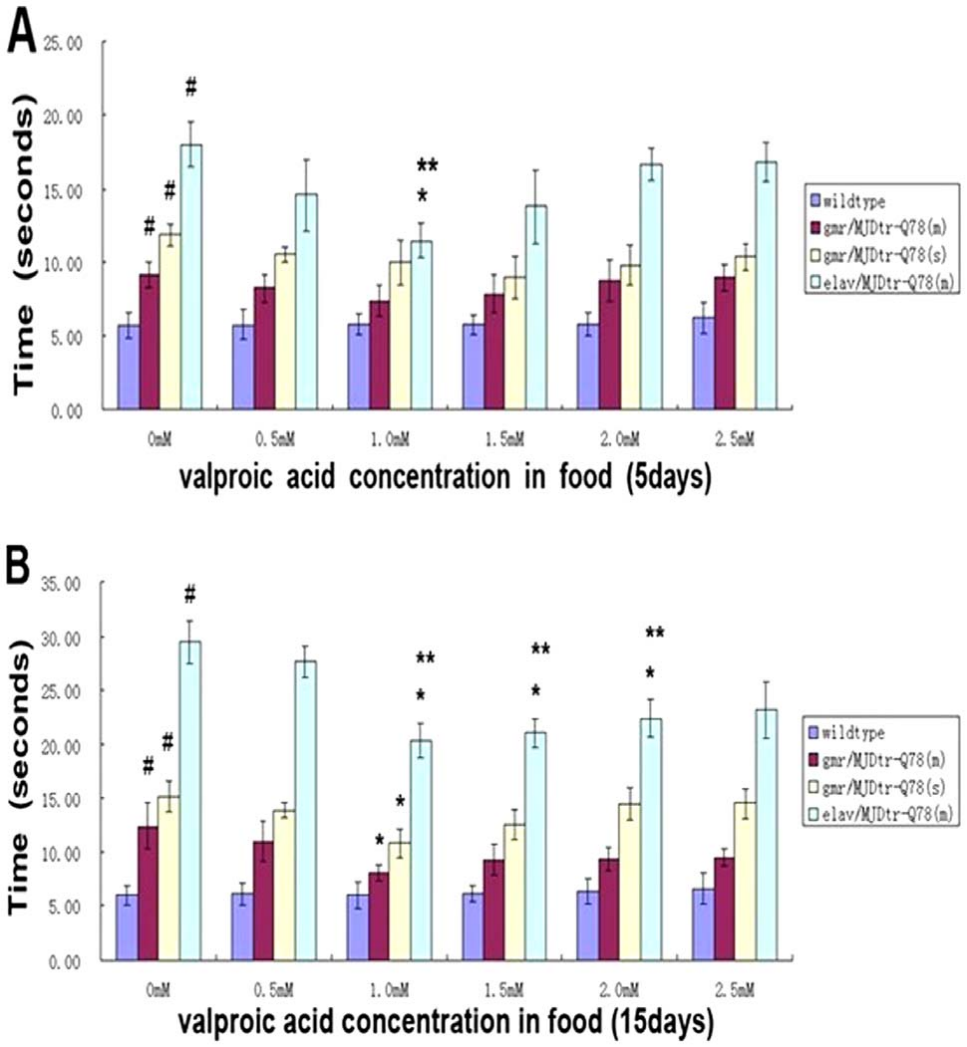
Valproic acid ameliorates climbing ability of SCA3 *Drosophila* model. Light-induced positive geotactic response (time to reach to 17.5 cm high) was investigated. Wild-type and MJDtr-Q78-expressing flies were tested following VPA treated for 5 (**A**) or 15 days (**B**) after flies reached adulthood, # represents compared to wild-type flies without treating with VPA, P<0.05; * represents compared to the same genotype flies without treating with VPA, P<0.05. ** Represents compared to the same genotype flies without treating with VPA, P<0.01. Error bars represent S.E.M (n = 200). Although wild-type flies showed no differences in behavior, all MJDtr-Q78-expressing flies showed a significant dose-dependent increase in performance, p<0.05. When VPA treated at doses from 0 mM to 1 mM, and the increase was obvious in *elav-MJDtr-Q78 (m)* than other genotypes. When VPA treated at a dose of 1.5 mM or higher, the climbing ability of all MJDtr-Q78-expressing flies were gradually reduced, even so, when VPA treated at doses of 2.5 mM, all MJDtr-Q78-expressing flies performed better than non-treated flies.

When treated with VPA at doses from 0 mM to 1 mM for 5 days, wide-type flies showed no differences in climbing ability compared to non-treated controls (data was not shown), MJDtr-Q78-expressing flies showed a dose-dependent increase in climbing ability, and the increase was greater for *ELAV-MJDtr-Q78 (m)* (Fig. 6A). However, when the dose reached to 1.5 mM or higher doses, both wide-type and MJDtr-Q78-expressing flies required more time to cross the line, suggesting a maximum therapeutic dose for clinical benefit after which climbing abilities may be further impaired. Similar results were found in flies with VPA treated for 15 days (Fig. 6B). The results showed that VPA treatment at a dose of 1.5 mM or higher did not positively benefit the SCA3 phenotype, proper dose for SCA3 *Drosophila* model may be from 0.5 mM to 1.5 mM.

### VPA elongates life span of MJDtr-Q78 transgenic *Drosophila*

**Figure 7:**
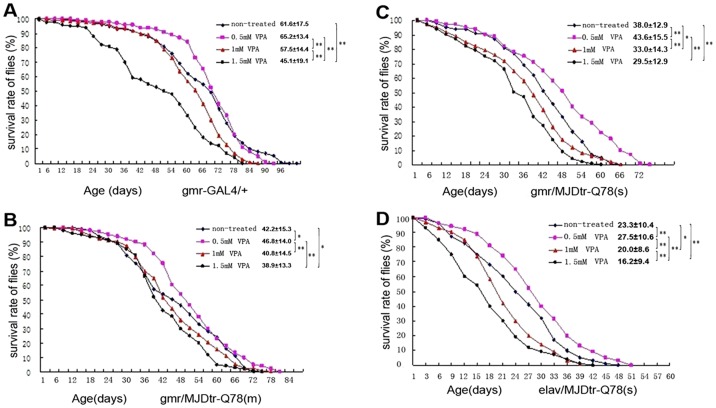
Valproic acid elongates mean lifespan of SCA3 *Drosophila* model. Longevity curves of wild-type flies (A), *gmr/MJDtr-Q78 (m)* (B), *gmr/MJDtr-Q78 (s)* (C) and *elav/MJDtr-Q78 (m)* (D) with VPA treated at 0 mM, 0.5 mM, 1.0 mM, 1.5 mM are shown in this figure. Life span shortening by moderate or strong expression of human mutant MJDtr-Q78 was rescued by VPA administered at a dose of 0.5 mM. When VPA doses were increased to 1 mM and 1.5 mM, the mean life spans of the three fly strains were shortened compared to those of the controls. The mean life span and SD are shown, *: p<0.05, **: p<0.01 (n = 100).

Because VPA was effective in preventing eye depigmentation and improving climbing ability in SCA3 *Drosophila* model, we next tested whether VPA affected the life span of SCA3 *Drosophila* model. As previous studies had showed that the proper dose of VPA for SCA3 *Drosophila* model was from 0.5 mM to 1.5 mM, we treated flies at doses of 0.5 mM, 1.0 mM and1.5 mM. As can be obviously seen in Fig.7-A, when VPA administered at a dose of 0.5 mM, the average lifespan and 10% survival time of wild-type flies showed no differences compared to non-treated flies (p>0.05), but as the dose gradually increased to 1.5 mM, the average life span shortened from the original mean of 61.7±17.5 to only 45.1±19.1 days. In contrast, after VPA was administered at a dose of 0.5 mM, the average life spans of *GMR-MJDtr-Q78 (m)*, *GMR-MJDtr-Q78 (s)* and *ELAV-MJDtr-Q78 (m)* flies were mildly prolonged; the average life span increased from the original means of 42.2±15.3, 38.0±12.9, and 23.3±10.4 days to 46.8±14.1, 43.6±15.5, and 27.5±10.6 days, respectively. When VPA doses were increased to 1 mM and 1.5 mM, the mean life spans of the three fly strains were shortened compared to those of the controls. The average life spans were described in Fig. 7-B, Fig. 7-C, and Fig. 7-D, respectively. The shortest mean longevity was observed at a dose of 1.5 mM. VPA at a certain dose (0.5 mM) thus had a positive effect on SCA3 pathology, but treatment at a dose of 1.0 mM or more caused obvious adverse effects.

### VPA does not inhibit mutant ataxin-3 protein aggregation

**Figure 8:**
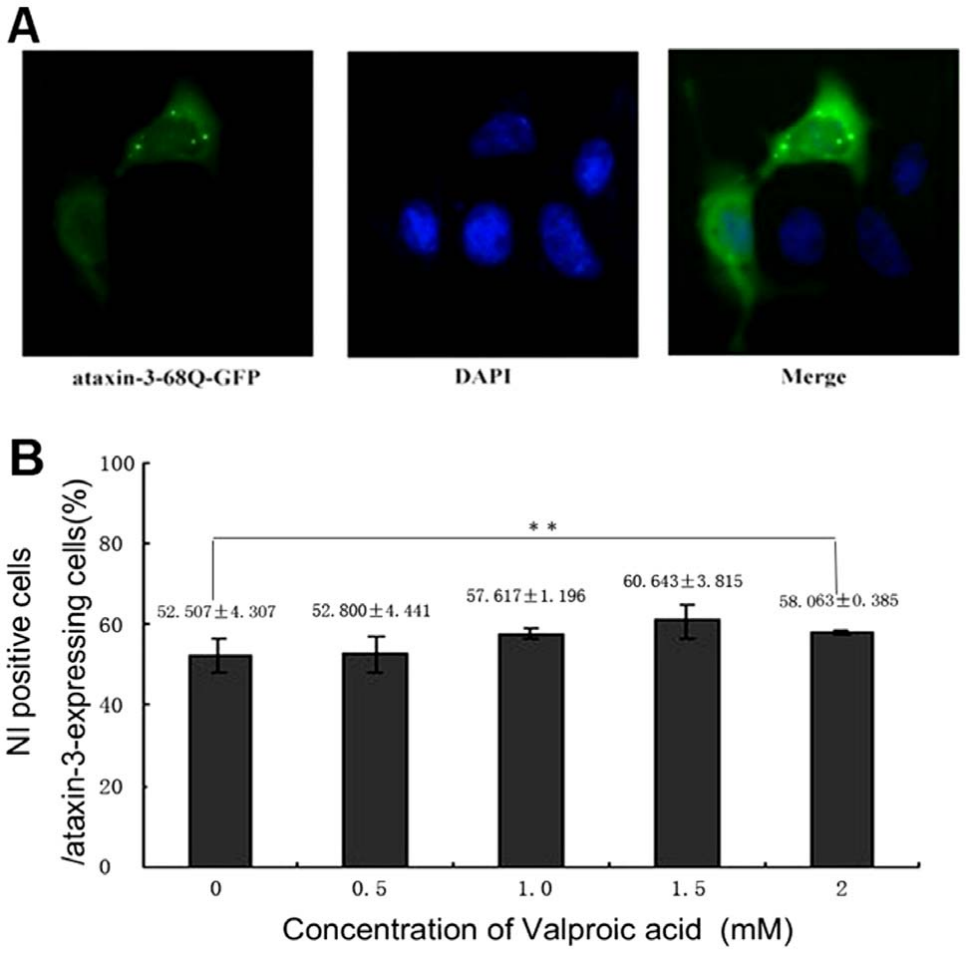
Valproic acid does not prevent aggregation of mutant Ataxin-3 protein. (**A**) Fluorescent microscopy showing the expression of ataxin-3-68Q-GFP protein (green) and DAPI stained nucleus (blue) in the same HEK293 T cells. In the picture, significant diffuse staining of ataxin-3 protein can be detectable both in the cytoplasm and in the nucleus, mutant ataxin-3(green)aggregates in transfected HEK293 T cells co-localize with nucleus (blue). (**B**) Statistical analysis demonstrated no differences in the amount of mutant ataxin-3 protein aggregations between VPA-treated and non-treated group, Error bars represent S.E (**: P>0.05).

To further confirm our results and extend our molecular analyses, we took advantage of a well-characterized cell culture model of SCA3. As can be seen in Fig. 8A, when HEK293T cells were transfected with pEGFP-N1-ataxin-3-68Q, they expressed a polyglutamine-expanded form of ataxin-3, which led to the appearance of nuclear inclusions in a substantial fraction (50%) of ataxin-3-expressing cells. In addition, significant diffuse staining of the ataxin-3 protein could be detectable both in the cytoplasm and in the nucleus. To rule out the possibility that VPA treatment reduced the amount of nuclear-localized mutant ataxin-3 protein aggregations *in vitro*, we used GFP fluorescence techniques to analyze the proportion of ataxin-3 aggregate- positive cells in ataxin-3-expressing cells at different doses of VPA intervention. As predicted, VPA did not alter the amount of nuclear-localized mutant ataxin-3 protein aggregations in ataxin-3-expressing cells (Fig. 8B). There were no significantly differences between the VPA-treated group and the controls (P>0.05).

### VPA increases acetylation levels of histone H3 and histone H4

**Figure 9:**
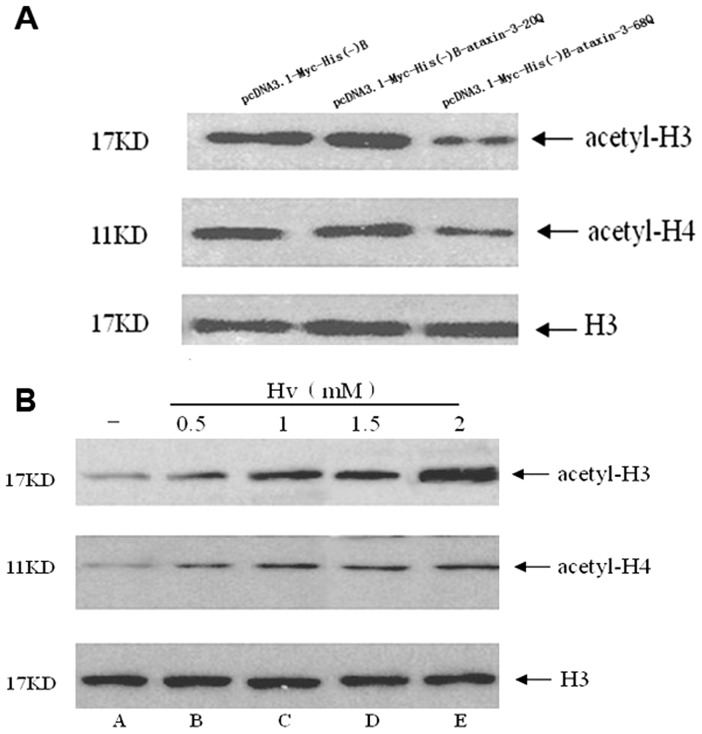
Valproic acid rescued the hypoacetylation of histone H3 and H4 *in vitro*. (**A**) Western blotting of acetylated histone H3 and H4 showed that wild-type and mutant ataxin-3 transfected cells and had different levels of histone acetylation. Mutant ataxin-3 protein led to hypoacetylation in transfected HEK293 T cells. Levels of H3 showed no differences between wild-type and mutant ataxin-3 transfected cells.The acetylation levels of histone H3, H4 were quantified using densitometry. (**B**) Different doses of VPA administration showed VPA increased the H3 and H4 histone acetylation level in a dose-dependent fation in SCA3 cell models. Levels of H3 showed no differences in cells expressing mutant ataxin-3 at 0.5 mM or higher doses.

If VPA did not function as an aggregation inhibitor, then the ameliorative effects of VPA treatment likely resulted from its ability to inhibit HDACs. We expected that the VPA treatment would have a direct effect on levels of histone acetylation. To test this hypothesis, we examined the acetylation levels of histones H3 and H4 by using antibodies specific to acetylated histones H3 and H4 and by using antibody against histones H3 as an internal control. Without VPA treatment, acetylation levels were reduced in cells expressing mutant ataxin-3 compared with wt (Fig. 9A). Prolonged VPA treatment resulted in a significant increase in the H3 and H4 histone acetylation levels in cells expressing mutant ataxin-3 at a dose of 0.5 mM or higher doses. This effect was significantly enhanced at a dose of 2 mM (Fig. 9B). In contrast to acetylated histones H3 and H4, the expression levels of histone H3 were not different in cells expressing mutant ataxin-3 at a dose of 0.5 mM or higher (Fig. 9B).

### VPA suppresses apoptosis in SCA3 cell culture model

**Figure 10:**
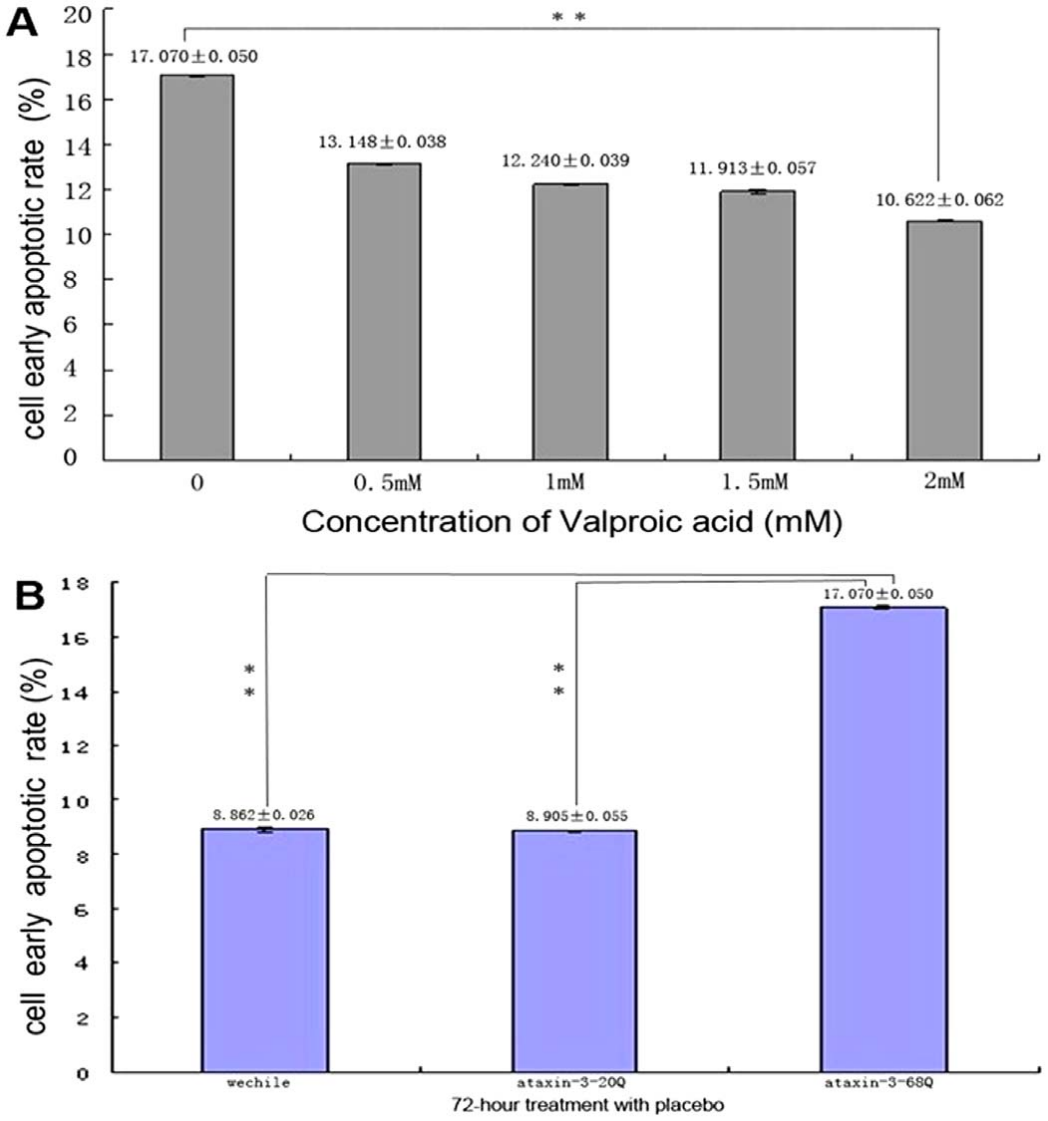
Valproic acid suppresses apoptosis in SCA3 cell culture model. (**A**) Apoptosis reduced by VPA was analyzed by flow cytometry analysis after staining with Annexin V and PI. Each data represents mean ± standard deviation.VPA treatment showed a dose-dependent reduction of early apoptotic rate, with increasing doses of VPA, the early apoptotic rate of ataxin-3-68Q cell line had gradually reduced from 17.07%±0.050% to 10.622%±0.062%, Error bars represent S.E, ^**^: P<0.05. (**B**) After 72-hour treatment with placebo, mutant ataxin-3 protein induced apoptosis in transfected HEK293 T cells, Error bars represent S.E, ^**^: P<0.05.

Although the molecular mechanisms underlying SCA3 have yet to be clarified, mounting evidences suggest that apoptosis, at least in part, is involved in the neurodegeneration [25–27]. VPA is an inhibitor of histone-deacetylases (HDAC) to regulate gene transcription and has been reported to suppress apoptosis by inducing the apoptosis-inhibiting gene *bcl-2* [20] and by inhibiting the mitochondria-mediated apoptosis pathway [21]. We predicted that VPA would have a direct effect on the apoptotic rate of SCA3 cell culture models. In this study, we used a flow cytometric analysis of Annexin V/propidium iodide binding to assess whether VPA treatment reduce the early cell apoptotic rate in three HEK293 T cell lines, each of which were transiently transfected with different ataxin-3 constructs. Consistent with previous reports, expression of these constructs in HEK293 T cells caused repeat length-dependent cell death. As can be seen in Fig. 10B, after a 72-hour treatment with placebo, cells expressing a normal-range repeat (20QP) ataxin-3 showed a similar early apoptotic rate (8.862%±0.026%) compared with the empty vector group (8.905%±0.055%) (P>0.05). Cells expressing an expanded repeat ataxin-3 (68QP) showed a higher early apoptotic rate (17.07%±0.050%) and there were significant differences between the mutant group and the controls (P<0.005) (Fig. 10B). To test whether VPA treatment showed the expected dose-dependent reduction of the early apoptotic rate, we treated the mutant ataxin-3-expressing cells with different doses (0.5 mM, 1 mM, 1.5 mM, 2 mM) of VPA and measured the early apoptotic rate. As can be seen from Fig. 10A, significant differences were found between each VPA-treated groups and the placebo-treated group. With increasing doses of VPA, the early apoptotic rate of the ataxin-3-68Q cell line had gradually reduced from 17.07%±0.050% to 10.622%±0.062%.

## Discussion

In this study, we demonstrated that VPA administered at an appropriate dose partly alleviated phenotypic markers of the ataxin-3 mutation in *Drosophila*. We also demonstrated that administration of VPA increased the acetylation levels of histone H3 and histone H4 and reduced the early cell apoptotic rate without inhibiting the aggregation of mutant ataxin-3 protein *in vitro*. These results were consistent with previous studies, in which HDAC inhibitors, such as SAHA [9] and butyrate [9], had been shown to reverse the hypoacetylation of histones H3, H4 *in vitro* and rescue polyQ-induced pathological effects in *Drosophila* models of HD. All these results suggested the significance of HDAC inhibitors, including VPA, as potential therapeutic agents for SCA3 and other polyQ diseases.

Here, a well-established *Drosophila* model of SCA3 was recreated. Besides its several neurodegenerative phenotypes that were similar to features of human SCA3, the *GMR-MJDtr-Q78* flies were characterized by the eye depigmentation as they should be, as well as a shortened life span and reduced climbing ability. Our finding suggested that *GMR-GAL4* should have a much more wide expression, rather than the eye-specific pattern. Although we did not perform western blots of different tissues to confirm this hypothesis, a previous study [28] in which a *UAS-LacZ* reporter gene driven by *GMR-GAL4* was found to express in third-instar larval wing discs, as well as in other tissues including brain, trachea and leg discs can address this possibility. Thus, more work should be done to confirm this idea, as they will certainly help the community at large for future studies.

The therapeutic effect of VPA in our SCA3 *Drosophila* model, that was VPA alleviated polyQ-induced phenotypic abnormalities in SCA3 *Drosophila* model when used at an appropriate dose, potentially provided us with a clinical prospect of HDAC inhibitors in the treatment of SCA3. Due to its narrow therapeutic window, these beneficial effects could be masked when VPA was used at higher doses. Similar results had also been shown in previous study [17] in which SB was given via oral administration to treat neurological phenotypes of SBMA, SB treatment within a narrow dose range improved motor impairment and survival rate and enhanced weight gain in the male Tg mice, whereas higher doses of SB caused deleterious effects. Since single agent possesses limited effects because of its narrow therapeutic window, in future studies combination of drugs seems to be an optimal attempt to maximize their therapeutic potency and minimize adverse effects.

To further confirm our results and to clarify the possible mechanisms of action of VPA in SCA3, We also tested therapeutic approaches for VPA using SCA3 cell models. In our study, VPA increased the histone acetylation levels of H3 and H4 in a dose-dependent fashion without inhibiting the aggregation of mutant ataxin-3 proteins. Previous studies had shown that histone acetylation and deacetylation could regulate a number of heat shock protein (HSP) gene transcriptions, including HSP22, HSP30 and HSP70 [29, 30]. HDAC inhibitors, such as TSA, BuA and VPA, could promote the expression of HSP70 and extend the life of wild-type flies [31–34], as well as evidence that over-expression of HSP70 slowed polyglutamine-induced neurodegeneration in SCA3 *Drosophila* model [35] and extended the lifespan of wild-type flies [36, 37]. Based upon this evidence, we infer that VPA acts as an HDAC inhibitor to restore the balance of histone acetylation levels, promote the expression of certain small molecule, such as HSP, and thus results in decreased neurodegeneration in SCA3.

In our study, we found that expression of different ataxin-3 constructs in HEK293 T cells caused repeat length-dependent cell death. Cells expressing an expanded repeat ataxin-3 (68Q) showed a higher early apoptotic rate compared with a normal-range repeat (20Q) group. Our results supported the hypothesis that mutant polyQ-containing proteins induced cell death *in vitro* and that apoptosis might underlie the polyQ diseases pathology. In our study, a decline in the early apoptotic rate was apparent in MJDtr-Q68-expressing cell when VPA was administered at 0.5 mM, and this effect was enhanced by increasing the dose. These findings were consistent with previous studies, which had shown that VPA could suppress apoptosis by inducing the apoptosis-inhibiting gene *bcl-2* [20] and by inhibiting the mitochondria-mediated apoptosis pathway [21]. In addition, Warrick [22] and Srimoyee Ghosh [38] found that overexpression of antiapoptotic gene *p35* successfully ameliorated retinal neurodegeneration in the *Drosophila* models of both SCA1 and SCA3, in combination with our study, suggesting that antiapoptosis may be one of the potential targets in polyQ-induced retinal cell death. Thus, we infer that VPA might play its role by suppressing retinal cell death via an antiapoptosis pathway.

In conclusion, our results suggest that VPA alleviates the phenotypes in SCA3 *Drosophila* model. According to our studies, at least two mechanisms may explain this neuroprotection. Firstly, VPA acts as an HDAC inhibitor and restores the imbalance in histone acetylation levels, thereby promoting the expression of certain small molecules, such as heat shock proteins. Secondly, VPA inhibits apoptosis via affection of certain signaling pathways. Therefore, VPA could be considered as a possible new therapeutic strategy for the treatment of SCA3 and other polyQ diseases, although its dose and duration need to be further explored clinically.

## Materials and Methods

### *Drosophila* stocks and crosses

All flies were raised on nutritional media containing mixture of cornmeal, glucose, sucrose, agar, 10% methylparaben and yeast powder. The growth incubators were maintained at 25°C and at 60%-70% relative humidity. The *w^1118^* strain was used as the background strain. The *UAS-MJDtr-Q27*, *UAS-MJDtr-Q78 (m)* and *UAS-MJDtr-Q78 (s)* [22] fly strains were obtained from the Bloomington *Drosophila* stock center. *GMR-GALl4* and *ELAV-GAL*4 were P{long*GMR-GAL4*}2 [39] and P{GawB}*ELAV^C155^* [40], respectively. They were received as gifts from the Chinese Academy of Sciences of Genetics and Developmental Biology research and from Dr. Xun-Hang. The expression of polyglutamine was driven by the bipartite expression system upstream activator sequence *UAS-GAL4* in transgenic flies. Constructs under the control of a yeast UAS were crossed with flies expressing the yeast GAL4 transcriptional activator, driven by the neuron-specific promoter *elav* and the eye-specific promoter *gmr*, which were selectively expressed in all neurons and in the developing eyes, respectively. The constructed genotypes were described as follows: P (*w;gmr-GAL4/+;UAS-MJDtr-Q27/+*), P (*w;gmr-GAL4/+; UAS-MJDtr-Q78(m)/+*), P (*w;gmr-GAL4/UAS-MJDtr-Q78(s);+/+*), P (*elav-GAL4/+;+/+;UAS-MJDtr-Q27/+*), P (*elav-GAL4/+;+/+;UAS-MJDtr-Q78(m)/+*), and P (*elav-GAL4/+;UAS-MJDtr- Q78(s)/+;+/+*). As flies of genotype *ELAV-MJDtr-Q78 (s)* exhibited the most serious neurodegenerative phenotype, no files of this phenotype survived to adulthood. We could only analyze SCA3 phenotypes in the remaining five genotypes.

### Drug administration

As VPA is known as a teratogen, we chose to administer this drug after the flies reached adulthood in order to avoid its side effect including developmental abnormalities. Before feeding files, VPA was prepared at 100 mM concentration in ion-free water and was added at appropriate concentrations in standard medium after it was cooled to below 50°C. To ensure the stability and efficacy of the VPA, the drug-containing medium was kept away from light and to be used right after it was ready. Flies were raised on standard medium at first and were placed into drug-containing medium since after eclosion from pupal cases.

### Eye morphology of flies

Heads were moved for electron microscopy from 15-day-old adult flies and fixed in 2.5% glutaraldehyde for 2 hr, followed by 1% osmic acid for 1 hr. They were then rinsed in PBS, dehydrated in serial dilutions of acetonum, and replaced by serial dilutions of isoamylacetate. Tissues were embedded in OCT (Tissue Tech) and frozen by immersion in liquid nitrogen. Adult eyes were viewed on a 1000 cx electron microscope (JSM-6490LV, Japan, Hitachi).

### Climbing ability

Ten flies were randomly selected from each sample and placed in a 25 ml pipette that was sealed at the top with wax film to prevent escape animals (n = 200 animals per group for each genotype). A fiber-optic lamp illuminated the pipette from the top. After adapting for 10 sec, the flies were gently knocked to the bottom of the pipette and the time required for the first 5 flies to cross a line 17.5 cm from the bottom was recorded. Five trials were completed for each sample. ANOVA and rank-sum tests were used to test the differences in climbing ability among groups. Data are presented as the mean±SEM.

### Plasmid constructs

The expression plasmids of ataxin-3 were previously constructed by Dr. Ya-Fang Zhou and Dr. Jiang-Guang Tang. The MJD1 cDNA in the plasmid was a truncated fragment including either 20 (normal, pEGFP-N1-20Q, pcDNA3.1-Myc-His (-) B-20Q) or 68 (expanded, pEGFP-N1-68Q, pcDNA3.1-Myc-His (-) B-68Q) repeats of CAG, which were inserted into the pEGFP-N1 vector (Invitrogen) and the pcDNA3.1 vector (Invitrogen), respectively.

### Cell culture and histone acetylation analysis

HEK293T cells, stably transfected with plasmids (Invitrogen) pEGFP-N1-20Q or pEGFP-N1-68Q, were cultured in DMEM containing 10% fetal bovine serum for 24 hr. They were treated for the following 48 hr with different doses (0.5 mM, 1.0 mM, 1.5 mM, 2.0 mM, 2.5 mM) of VPA. Controls were uninduced cells. Cells were lysed in RIPA buffer supplemented with a complete protease inhibitor cocktail (Roche, Branchburg, NJ), and protein lysate was quantified using a BCA kit (Bio-Rad). Protein content was fractionated on a 12% SDS-PAGE gel and transferred to a protran nitrocellulose membrane (Amersham). Membranes were blocked for 1 hr at room temperature in 3% BSA containing 5% non-fat dried milk. Next, membranes were incubated with primary antibodies in 3% BSA containing 1% non-fat dried milk with gentle agitation for 1 hr at 4°C. The following primary antibodies were used for western blot analysis: anti-acetylated histone H4 (1∶1000), anti-histone H3 (1∶1000) and anti-acetylated histone H3 (1∶1000) (all from Upstate Biotechnology). Signals were detected by the enhanced chemiluminescence western blot detection system (Amersham Biosciences, GE Health Care Biosciences, Hong Kong).

### Detection of apoptosis in SCA3 cell culture model

Cell cultures were produced by transfecting plasmids (Invitrogen) pcDNA3.1- Myc-His (-) B-20Q or pcDNA3.1-Myc-His (-) B-68Q into HEK293T cells. Apoptotic cells were detected using PI/Annexin V-FITC double staining flow cytometry (Becton-Dickinson). Briefly, cells were untreated or treated with different doses of VPA (0.5 mM, 1.0 mM, 1.5 mM, 2.0 mM, 2.5 mM) for 48 hr at 37°C. The cells were trypsinized and washed with PBS, then resuspended in Annexin-V binding buffer at 1×10^6^/ml. The cells were stained simultaneously with FITC-conjugated Annexin-V and PI at room temperature for 15 min in the dark, following by the addition of binding buffer. The apoptotic cells were measured using a FACScan flow cytometer. The cells were sorted into intact cells (Annexin V− PI−), early apoptotic cells (Annexin V+ PI−), late apoptotic cells (Annexin V+ PI+), and necrotic cells (Annexin V− PI+). Three trials were completed for each sample. Student's t-test and ANOVA were used to test the differences in early apoptotic rates among groups. Data are presented as the mean±SEM.
